# Critical hydrodynamic force levels for efficient removal of oral biofilms in simulated interdental spaces

**DOI:** 10.1007/s00784-024-05739-7

**Published:** 2024-05-31

**Authors:** Merima Hotic, Mario Ackermann, Joshua Bopp, Norbert Hofmann, Lamprini Karygianni, Pune Nina Paqué

**Affiliations:** 1 School of Engineering, Institute of Thermal and Fluid Engineering, University of Applied Sciences Northwestern Switzerland, Windisch, Switzerland; 2 University of Applied Sciences Northwestern Switzerland, Windisch, Switzerland; 3 Center for Dental Medicine, University of Zurich, Clinic for Conservative and Preventive Dentistry, Zurich, Switzerland; 4 Center for Dental Medicine, University of Zurich, Clinic for Reconstructive Dentistry, Plattenstrasse 11, Zurich, CH-8032 Switzerland

## Abstract

**Objectives:**

Sonic toothbrushes generate hydrodynamic shear forces for oral biofilm removal on tooth surfaces, but the effective thresholds for biofilm removal remain unexplored. This in vitro study aimed to investigate various threshold values for hydrodynamic biofilm removal in vitro.

**Materials and methods:**

A specialized test bench was designed with a known water flow field within a gap, ensuring that hydrodynamic shear forces on the wall were solely dependent on the volume flow, which was quantifiable using an integrated flow meter and proven by a computational fluid dynamics simulation. A young 20 h supragingival six-species biofilm was developed on hydroxyapatite disks (∅ 5 mm) and applied into the test bench, subjecting them to ascending force levels ranging from 0 to 135 Pa. The remaining biofilms were quantified using colony forming units (CFU) and subjected to statistical analysis through one-way ANOVA.

**Results:**

Volume flow measures < 0.1 l/s: Error 1% of reading were established with the test bench. Untreated biofilms (0 Pa, no hydrodynamic shear forces) reached 7.7E7 CFU/harvest and differed significantly from all treated biofilm groups. CFU reductions of up to 2.3E6 were detected using 20 Pa, and reductions of two orders of magnitude were reached above wall shear forces of 45 Pa (6.9E5).

**Conclusions:**

Critical hydrodynamic force levels of at least 20 Pa appear to be necessary to have a discernible impact on initial biofilm removal.

**Clinical relevance:**

Pure hydrodynamic forces alone are insufficient for adequate biofilm removal. The addition of antiseptics is essential to penetrate and disrupt hydrodynamically loosened biofilm structures effectively.

**Supplementary Information:**

The online version contains supplementary material available at 10.1007/s00784-024-05739-7.

## Introduction

The etiology of prevalent oral diseases such as caries and periodontitis are rooted in the complex and dynamic interactions between oral bacteria and oral environment of the host [3, 11]. A central element in the development of these diseases is the accumulation of biofilms, which are well-organized communities of microorganisms embedded within a matrix of extracellular polymeric substances (EPS) [10]. These biofilms adhere to tooth surfaces and oral soft tissues, creating an environment conducive to the production of acids and inflammation, ultimately leading to caries, periodontitis, and other oral diseases [17].

To combat these oral diseases and maintain optimal oral health, a wide array of oral hygiene devices is employed by individuals worldwide [2, 16]. Among these, toothbrushes are one of the most universally used tools [5]. The effectiveness of toothbrushes in biofilm removal is based on several key mechanisms [4, 6]. First, there are the mechanical actions of the bristles, which physically disrupt and dislodge biofilms from tooth surfaces [13, 20]. Second, the properties of the toothpaste slurry, including its abrasive nature and chemical components, play a role in breaking down and removing biofilms [14, 15]; [1]. Lastly, the hydrodynamic forces generated during tooth brushing, such as fluid shear and turbulence, contribute significantly to biofilm disruption by carrying away biofilm particles [9].

In recent years, sonic toothbrushes have gained significant popularity for their capacity to harness hydrodynamic forces in biofilm elimination [25, 28]. These devices operate at high frequencies, producing rapid movements of the bristle tips and generating dynamic fluid flows within the oral cavity. This dynamic action enhances the removal of biofilms, especially in hard-to-reach areas, such as the interdental sites [6, 12, 27].

The effectiveness of sonic toothbrushes in mechanically removing biofilms through direct contact has been the subject of extensive investigation. Studies have evaluated their ability to reduce plaque accumulation and improve overall oral hygiene [18, 21]. However, it is essential to recognize that not all tooth surfaces and oral structures are easily accessible to the bristles of toothbrushes, be they manual or sonic. Proximate areas between teeth and tight interdental spaces can pose a challenge for traditional brushing methods [13]. In these challenging areas, the synergy between hydrodynamic forces and the properties of toothpaste slurry becomes especially relevant. The dynamic fluid movements generated by sonic toothbrushes, along with the action of toothpaste, play a crucial role in biofilm removal from these areas. Yet, despite the importance of these non-contact brushing methods, the establishment of threshold values for hydrodynamic forces required to effectively remove oral biofilms remains a relatively underexplored aspect of oral hygiene research [19].

The existence of such thresholds is of utmost importance for refining oral hygiene practices and enhancing biofilm control strategies. By gaining a deeper understanding of the minimum force required for effective biofilm removal, we can optimize the design and usage of oral hygiene devices. This knowledge can lead to improved oral health outcomes and a reduced risk of dental diseases.

The primary objective of this in vitro study is to address this critical knowledge gap. We are conducting a comprehensive analysis of different threshold values for hydrodynamic biofilm removal in vitro, focusing on simulated proximal tooth surfaces. Our research aims to provide precise insights into the amount of hydrodynamic force needed to achieve effective biofilm removal in these challenging areas. By shedding light on these thresholds, we hope to contribute valuable information that can inform the development of more effective oral hygiene practices and devices, ultimately enhancing biofilm control and promoting better oral health for individuals worldwide.

## Materials and methods

### Test bench

**Fig. 1:**
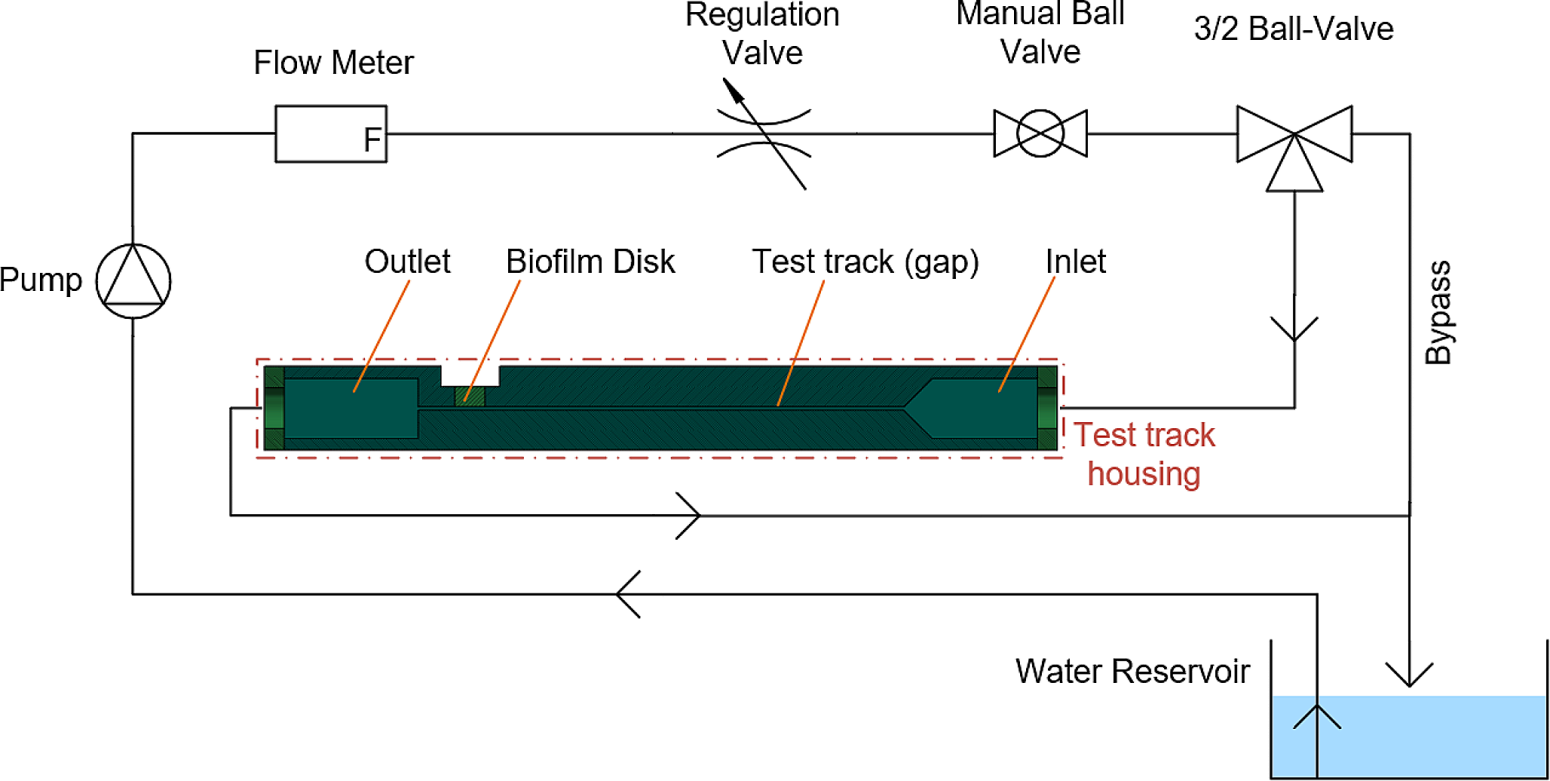
Schematic representation of the test bench

The test bench which is used to determine the threshold value for effective biofilm removal with hydrodynamic shear forces was developed during the Innosuisse project with Curaden AG (Innosuisse grant No 45807.1). Figure 1 shows the schematic representation of the test bench. The main component of the test bench is the test track housing which consist of the inlet nozzle, a test track (gap), the support for the biofilm disk and the outlet. The test track has a rectangular cross section with a gap of 2 mm height and 40 mm width. The distance between the inlet nozzle and the biofilm disk is 250 mm. Within this gap, the flow field of water is well known so that the hydrodynamic shear forces at the wall only depend on the volume flow. The necessary volume flow is measured ahead of the test track.

The flow meter, used to measure the water volume flow is an electromagnetic flow measuring system (Promag A, Endress + Hauser AG, Reinach, Switzerland). The measuring device has a display which shows the current value in liters/second. The nominal diameter of the device is 15 mm. According to the datasheet of the manufacturer, the measuring error depends on the volume flow and is presented below:

Volume flow < 0.1 l/s: Error 1% of reading.

Volume flow > 0.1 l/s: Error 0.5% of reading.

### Test bench operation

Water from the reservoir is pumped through the flow meter and the three valves. At the 3/2 ball-valve, the flow path is switched between bypass mode (direct to the reservoir) or test mode (through the test track). The regulation valve allows for setting the required water volume flow. Based on the geometry of the test track and the volume flow, the Reynolds number is within the range of 2’500 to 16’000 at the test conditions. For this reason, a turbulent flow can be assumed here. The Reynolds number is used to determine the flow regime and is calculated as presented below where $\rho$ is the density of the fluid, $w$ the flow velocity, $D$ the height of the test track and $\mu$ the fluid viscosity. The higher this number, the more chaotic and more irregular the flow pattern is and, the higher the hydrodynamic shear stress at the wall.

$$Re=\frac{\rho w D}{\mu }$$

### Wall shear stress measurements

**Fig. 2:**
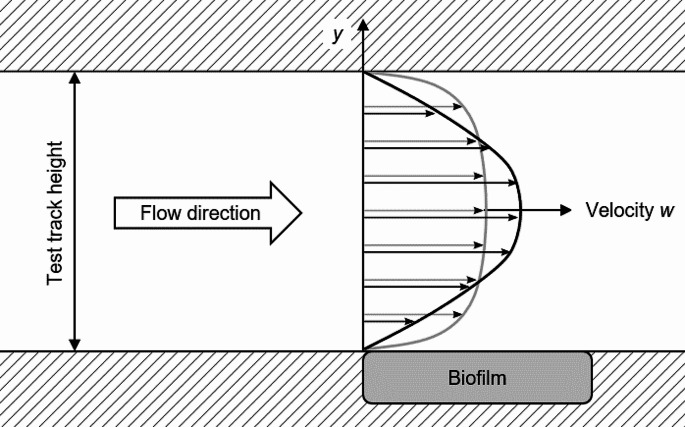
Velocity profile within the test track. Comparison between a low and a high velocity

As the shear stress on the test track is of interest, a detailed view of the test track with the biofilm disc is shown in Fig. 2. This represents the experimental application where the biofilm disk is mounted within the lower wall of the test track. The direction of the fluid flow is determined with the arrow (from left to right). At the wall, the fluid velocity is zero. The region of interest over the biofilm disk experiences a force along the flow direction because of the increasing velocity apart from the wall. The force is even higher, the faster the velocity increases. Figure 2 shows two velocity profiles within the test track. The profile in grey with the higher velocity increase at the wall also induces higher wall shear stresses, although its velocity in the center is lower compared to the velocity profile in black.

**Fig. 3:**
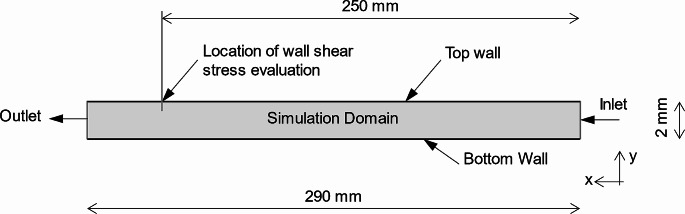
Simulation domain of the test track with the boundaries and coordinate directions x and y

At the test rig, the wall shear stress may not be measured directly. Therefore, the measurement of the volume flow is used to evaluate the wall shear stress. To determine the correlation between the measured volume flow and the wall shear stress, a computational fluid dynamics simulation was performed. The flow within the test track was simulated under equal conditions as at the test rig and the geometry corresponds to the test track gap as shown in Fig. 3. At the location where the biofilm disk is located on the test track, the wall shear stress is evaluated in the simulation. The simulation domain is modeled with a regular mesh composed of cells. At the center of each cell, the state variables are computed iteratively by the simulation tool. The basic mesh has a maximum cell size of 0.15 mm. To resolve the boundary layer the mesh is refined with hexaedrons at the top and bottom wall. The thickness of these hexaedron layer is 0.6 mm with a number of 27 layers and a growth rate of 1.2. The boundaries of the simulation are shown in Fig. 3. At the inlet a fixed velocity is defined, and the outlet is defined as an opening to ambient conditions. The top and bottom wall are specified as no-slip walls.

As mentioned in Sect. 2.2, the flow within the test track is turbulent which requires an adequate simulation model. The k-Omega turbulence model is widely used in engineering applications where the near-wall flow field is of interest. According to Wilcox [29] La Canada, CA: DCW industries.) for computational fluid dynamics (CFD) simulation the turbulence model k-Omega is used for wall-bound shear flows. The evaluation of the wall shear stress *t*_w_ is presented below.

$${\tau }_{w}=\mu {\left(\frac{dw}{dy}\right)}_{y=0}$$

The viscosity of the fluid *µ* is a material property and has the unit [Pa s]. The derivative $\frac{dw}{dy}$ represents the vertical velocity gradient at the wall.

### Inoculum for biofilm formation

The inoculum for the biofilm experiments was composed of *Actinomyces oris* OMZ 745, *Candida albicans* OMZ 110, *Veillonellea dispar* OMZ 493, *Fusobacterium nucleatum* OMZ 598, *Streptococcus oralis* OMZ 607, and *Streptococcus mutans* OMZ 918. The six strains were cultivated on Columbia blood agar plates (CBA, bioMérieux, Petit-Lancy, CH), enriched with 5% (v/v) sheep blood, under anaerobic conditions at 37 °C for at least 3 days. The Candida strain was cultivated under aerobic conditions (10% CO_2_). The microorganisms were isolated from the CBA plates and inoculated into 4 ml of mFUM universal fluid media (FUM) + 0.3% glucose supplemented with Sørensen’s buffer at a final of pH 7.2) and further incubated anaerobically at 37 °C. In case of *V. dispar*, the medium supplemented with 1% Na-lactate. The media of *A. oris, S. oralis,* and *S. mutans* were supplemented 1:2 with modified brain heart infusion (BHI + 5 µg/ml Hemin + 1 µg/ml Menadione).

After 24 h of incubation, the microbial suspensions of the single strains were adjusted to an optical density of OD_550_ = 1.0 +/- 0.05 using fresh mFUM and combined in equal proportions to create the inoculum.

### Collection of Saliva and Pellicle formation

Whole saliva of volunteers was collected, frozen, later thawed, pooled, and centrifuged to produce processed saliva. After pooling and centrifugation (30 min, 4 °C, 27,500 x g), the supernatant was pasteurized (30 min, 60 °C) and centrifuged again. The resulting supernatant was stored at -20 °C. For pellicle formation, hydroxyapatite discs (HA; Ø 5 mm; Clarkson Chromatography Products Inc., PA, USA) were placed in 6-well polystyrene plates and covered with 4 ml of processed saliva diluted 1:2 with 0.25% NaCl and sterile-filtrated (0.02 μm) for 4 h.

### Oral biofilm formation

The formation of the biofilms was carried out following established procedures, as previously described in detail [10, 26]. For these experiments, a newly developed young 20 h supragingival six-species biofilm was used. In brief, 500 µl of the inoculum were inoculated into 4000 µl of the preconditioned medium (composed of 1.2 ml mFUM mixed with 2.8 ml processed diluted saliva and anaerobically incubated at 37 °C with the pellicle-preconditioned HA disks) [8]. The carbohydrate concentration of mFUM was changed with the medium change after 3 h from 0.3% glucose to 0.15% glucose and 0.15% sucrose. After 17 h, a young biofilm formation was reached, and the HA disks were washed three times in 0.9% NaCl to remove loosely attached microorganisms. The biofilm-coated HA disks were stored in 6-well polystyrene plates covered with 0.9% NaCl and transported on ice to the test bench experiments immediately.

#### Pretesting of the young biofilm

The young biofilm model was pretested on 82 hydroxyapatite disks prior to test bench interventions. The cfu analysis was performed on these biofilms for quantifications as described below, and a visual analysis was performed using clsm (Leica Stellaris 5 inverse, Leica Microsystem, Wetzlar, Germany), equipped with an Leica white light laser after live/dead staining (*Bac*Light^™^, SYTO 9/PI, Invitrogen Ltd., Paisley, UK) according to the manufacturer’s instructions [23]. The stained biofilm disks were visually analyzed using a x63/1.4 NA oil-immersion objective lens and 1.7 digital magnification mode, with excitation/emission settings for SYTO 9 of 496 nm/500–545 nm and for PI of 549 nm/580–720 nm using two separate hybrid (Power HyD S) detectors and emission detection by spectral detectors. The data were subjected to the software Imaris (Version 10.0.0, Bitplane, Zurich, Switzerland).

### Treatment with different thresholds

The biofilm-coated HA disks were carefully placed into the test bench using tweezers. The biofilm-coated surfaces were positioned towards the flow path. The flow meter was set to 5 different settings with *n* = 4 biofilm disks per setting, and per cycle (135 Pa, 90 Pa, 45 Pa, 20 Pa, and 5 Pa). Biofilm controls (*n* = 4) were used without the test bench intervention and referred to as 0 Pa. The measurements were repeated in a second cycle with identical settings and numbers. Each flow rate was applied for 10 s. The flow-treated biofilm disks were then removed carefully from the test bench and subjected to the harvesting procedures. The cleaning of the test bench was performed with the use of a disinfectant solution, which was applied daily between the cycles (Sanosol S015, Sanosil AG, Hombrechtikon, Switzerland) and afterwards rinsed again with tap water.

### Harvesting with colony forming units

Following the experimental intervention in the test bench, the biofilm disks underwent a thorough cleansing process involving three rinses with 0.9% NaCl to eliminate non-adherent microorganisms. To collect the biofilms, the disks were transferred into.

500 µl of 0.9% NaCl, subjected to a 2-minute vortexing procedure, after which the disks were carefully removed. During this process, all other biofilms disks were stored and shaked on a 6° C cooled thermomixer at 2000 rpm (Thermomixer C, Eppendorf, Schönenbuch, CH). The suspensions were then briefly sonicated for 5 s on ice at 30 W using a Sonifier (B-12 Branson Ultrasonic located in Urdorf, Switzerland). Subsequently, the resulting microbial suspensions were subjected to serial dilutions in 0.9% NaCl and plated on non-selective agar plates Columbia blood agar plates, (CBA, bioMérieux, Petit-Lancy, CH), enriched with 5% (v/v) sheep blood to quantify the colony-forming units (CFU). 5 µl of the concentrated microbial suspension and of serial dilutions (10^− 1^, 10^− 2^, 10^− 3^, 10^− 4^, 10^− 5^) were pipetted onto the plates in duplets and incubated for a duration of 4 days under anaerobic conditions.

### Statical analysis

The metric variables of the remaining biofilms were analyzed as cfu harvest/disk and are further defined as cfu/disk. The quantified data were characterized using descriptive statistics including mean, median, standard deviations, quartiles, minimum, and maximum values (Table). Data analysis involved the application of ordinary one-way analysis of variance (ANOVA) to assess disparities in biofilms following treatment with increasing shear stress levels. Tukey’s multiple comparisons test was applied for post hoc corrections. Statistical analysis was conducted using GraphPad Prism software (version 10.1.1; Boston, MA, USA), with a significance level set at *p* < 0.05.

## Results

### Thresholds

**Fig. 4:**
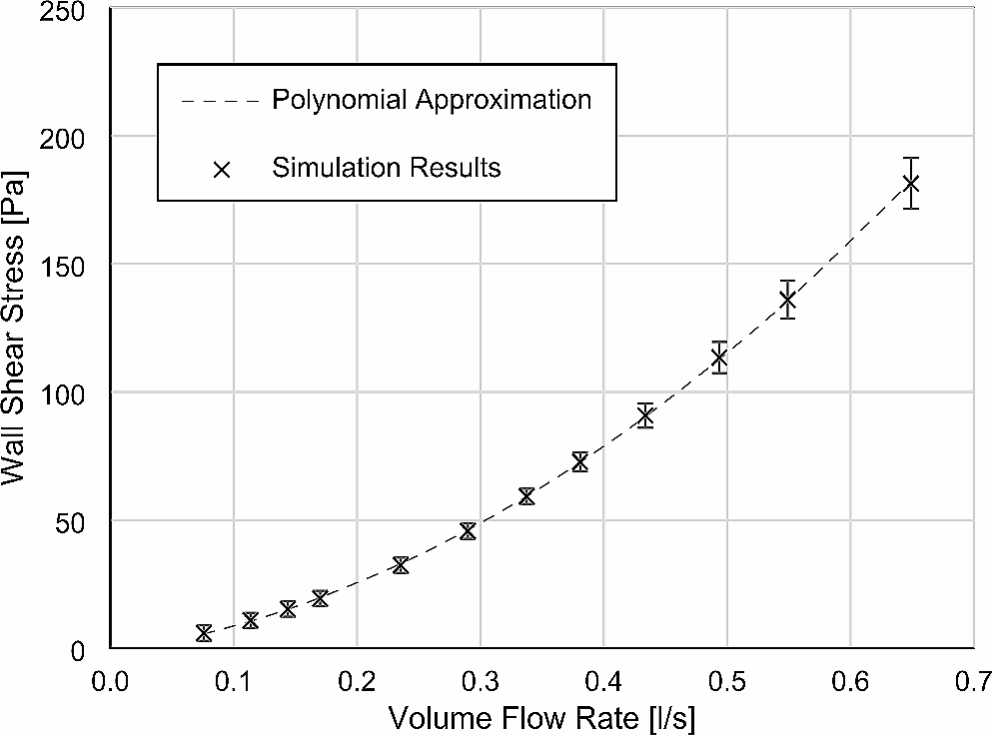
Corelation of the volume flow within the test track and the wall shear stress at the biofilm location. Simulation results with error bars and a polynomial approximation (y[Pa] = a x^2^ + b x + c, a = 328.541, b = 69.75, c = -1.67, x in [l/s])

Based on the simulation carried out, it was possible to correlate the volume flow with the wall shear stress. Figure 4 displays the simulation output with error bars. With this computational fluid dynamics simulation and the measurement of the volume flow, the wall shear stress is determined with a relative error of less than 10% above 20 Pa wall shear stress. At wall shear stress values < 20 Pa, the error increases to 20%.

### Young biofilm validation

**Fig. 5:**
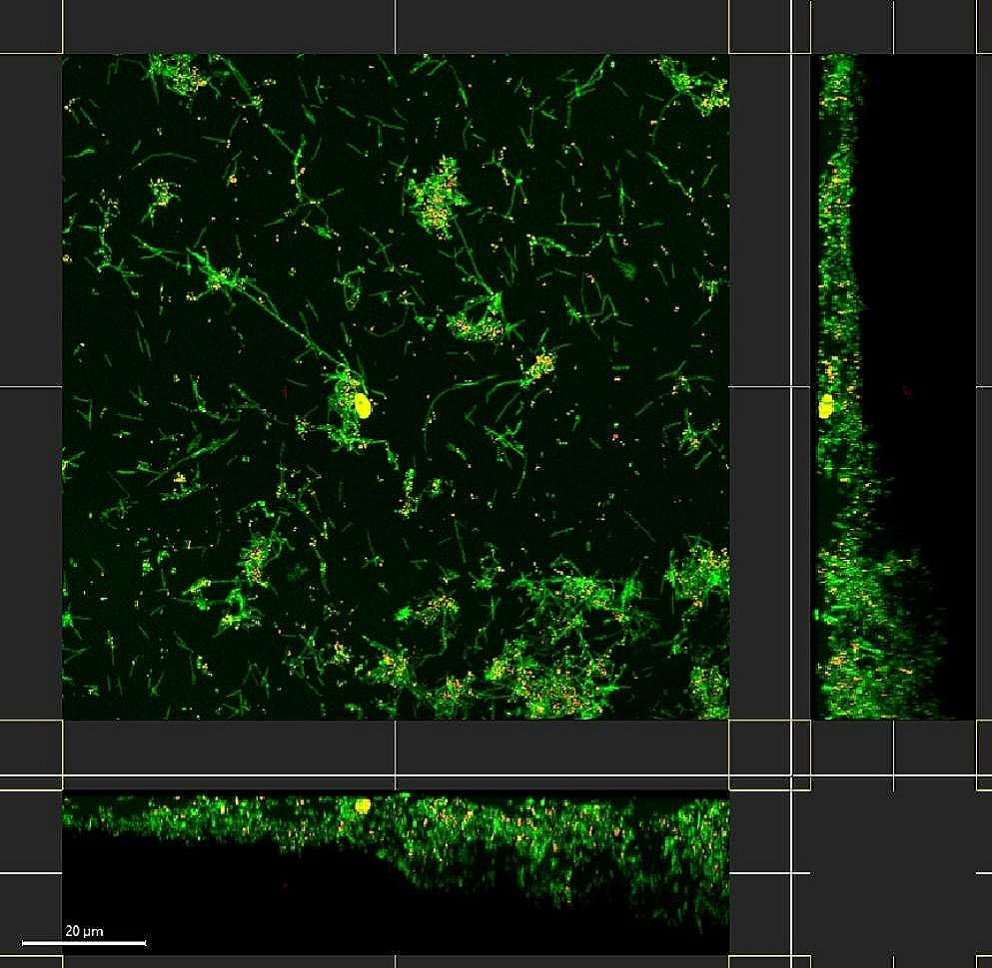
3D-Visualization of the young 20 h biofilm using clsm data after fluorescent live/dead staining with a scale bar of 20 μm. Viable cells are stained with Syto 9 (green) and cells with higher membrane permeability are stained with propidium iodide (red). The cross section of the biofilm shows loosely dispersed cells, with multiple accumulated microorganisms in coaggregation

A total of 82 disks were analyzed in 15 separate runs during the pretesting of the experiments, resulting in median cfu counts of 1.3E7 (IQR 3.3E7). The visualization with clsm shows the young biofilm adherence on the biofilm disk with different viability conditions, according to the permeability to the stains (Fig. 5). In Fig. 5, multiple coaggregations of microorganisms are evident, indicating an increase in density, whereas larger areas of the disks exhibit only thinner monolayers. By the 20-hour mark, the predominantly viable cells have progressed to a stage of coaggregation, fostering increased cellular contacts and proximity.

### Quantification of the remaining biofilm

The median, average, and standard deviation data are presented in a Table. Ascending force values led to ascending biofilm reduction. The median untreated control biofilm (0 Pa) resulted in 7.7E7 cfu/disk. The untreated controls and the 5 Pa shear force differed significantly from all other treated biofilm groups. 20 Pa differed significantly from the 90 Pa and 135 Pa groups, and 45 Pa differed significantly to the 135 Pa group.

**Fig. 6:**
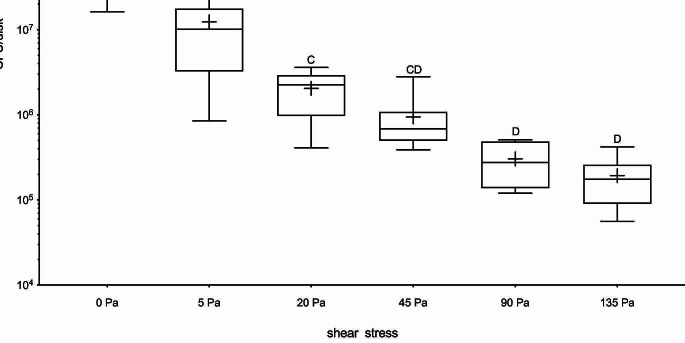
Boxplots with 25th and 75th percentiles, median, and mean showing the remaining biofilm as harvested CFU after intervention for 10 s with ascending force levels (0–135 Pa). Significant differences between the groups are marked with different letters

Biofilm reduction with 5 Pa resulted in median cfu counts of 1.0E7, and with 20 Pa in 2.3E6. cfu reduction of a magnitude of two were only reached above forces of 45 Pa. The maximum biofilm removal was measured, when 135 Pa were applied, resulting in cfu counts of 1.8E5. Figure 6 shows the boxplots and all mean, standard deviation (SD), median, and IQR data are presented in Table 1.

**Table 1:** Descriptive mean values with standard deviation (SD), median, and interquartile ranges (IQR) of the biofilm-coated disks after treatment with ascending forces for 10 s (*n* = 8 HA disks per group)

Group	0 Pa	5 Pa	20 Pa	45 Pa	90 Pa	135 Pa
Mean	9.4E7	1.2E7	2.0E6	9.5E5	3.0E5	1.9E5
SD	1.0E8	1.2E7	1.1E6	7.9E5	1.7E5	1.2E5
Median	7.7E7	1.0E7	2.3E6	6.9E5	2.8E5	1.8E5
IQR	4.9E7	7.7E6	1.6E6	2.8E5	3.2E5	1.1E5

## Discussion

The aim of this project was to find out how high the shear stress near the wall, i.e. on the tooth, must be to be able to remove a statistically relevant amount of adherent biofilm. For this reason, shear stresses of up to 135 Pa were set in steps of 45 Pa on a constructed test stand to characterise and classify the behaviour of the young biofilm.

Quantification of the remaining biofilm demonstrated that higher shear stress values induced a higher biofilm removal on the HA disks. Interestingly, force levels of 45 Pa were needed to show biofilm removal in 2 orders of magnitude and 1 order of magnitude if 20 Pa force levels were applied. Force levels of up to 135 Pa resulted still in a high number of remaining biofilms as calculated by cfu counts. A recent study on biofilm removal from water distribution systems indicated that high hydrodynamic forces of 135 Pa are needed to remove biofilms completely. However, these tests were based on a dead-end model with a single-species biofilm of *Escherichia coli* [22].

The clinical efficacy of oral devices that generate hydrodynamic shear forces, such as sonic toothbrushes and oral irrigators, has been demonstrated. It should be noted, however, that while higher shear forces, particularly with oral irrigators, enhance biofilm removal, complete biofilm removal remains unproven [7, 24].

For these experiments, we developed a young multi-species 20 h biofilm to mimic tooth surfaces before daily oral hygiene measures. The multi-species character of the biofilm depicts the complex biofilm architecture and was tested in previous settings over the past decades as more mature bench biofilm before [26].

The test rig was designed that high forces could directly affect the biofilm.

As mentioned in Chap. 2.3, the viscosity of the fluid depends on the temperature and therefore directly impacts the wall shear stress. If the fluid temperature fluctuates between 18 and 25 °C, the viscosity of water lies in a range between 1.05 and 0.89 mPa s. During the tests, care was taken to ensure that the fluid temperature fluctuates by a maximum of 2 °C. However, the main influence on the uncertainty of the wall shear stress are the meshing parameters and the turbulence model of the simulation. At least five cells were used to resolve the boundary layer closest to the wall (yPlus < 1), which leads to a reliable resolution of the velocity profile at the wall. Additionally, a mesh study was performed where all meshing parameters were examined and considered in the uncertainty analysis. In the third dimension (z coordinate in Fig. 3), the basic mesh has a thickness of only 0.1 mm, which corresponds to a two-dimensional simulation domain. The influence of a three-dimensional mesh with the equal extent as the test track of 40 mm in the z-coordinate is also considered in the uncertainty analysis. The results of the three-dimensional simulation were slightly below the two-dimensional results because of velocity variations along the z coordinate. Considering the viscosity, the meshing parameters and the turbulence model of the simulation, an uncertainty of less than 10% at values > 20 Pa results, which is adequate enough for the application presented.

## Conclusions

Hydrodynamic forces must reach a critical threshold of at least 20 Pa to noticeably affect the removal of an initial biofilm. However, relying solely on hydrodynamic forces is inadequate for effectively removing biofilm. It remains crucial to introduce antiseptics to effectively penetrate and disrupt the biofilm structures loosened by hydrodynamics.

### Electronic supplementary material

Below is the link to the electronic supplementary material.

Supplementary Material 1

Supplementary Material 2

## Data Availability

All data are presented in the manuscript.
