# Supplementary figures and images for: Critical hydrodynamic force levels for efficient removal of oral biofilms in simulated interdental spaces
Source: Clin Oral Investig. 2024 May 31;28(6):346. doi: 10.1007/s00784-024-05739-7 (PMC11142948; doi:10.1007/s00784-024-05739-7)

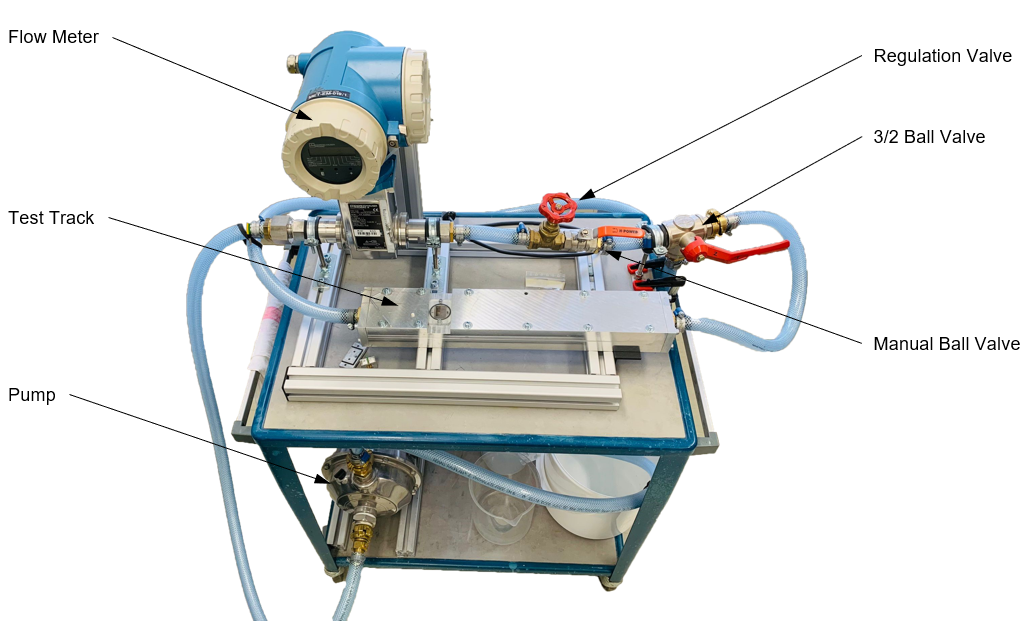

Supplement: Supplementary file 1 — Supplementary Material 1 [file 784_2024_5739_MOESM1_ESM.tif]
